# Lateralization of spatial information processing in response monitoring

**DOI:** 10.3389/fpsyg.2014.00022

**Published:** 2014-01-24

**Authors:** Ann-Kathrin Stock, Christian Beste

**Affiliations:** Cognitive Neurophysiology, Department of Child and Adolescent Psychiatry, TU DresdenDresden, Germany

**Keywords:** Simon task, response monitoring, spatial congruency, response evaluation, EEG, multisensory integration, proprioception

## Abstract

The current study aims at identifying how lateralized multisensory spatial information processing affects response monitoring and action control. In a previous study, we investigated multimodal sensory integration in response monitoring processes using a Simon task. Behavioral and neurophysiologic results suggested that different aspects of response monitoring are asymmetrically and independently allocated to the hemispheres: while efference-copy-based information on the motor execution of the task is further processed in the hemisphere that originally generated the motor command, proprioception-based spatial information is processed in the hemisphere contralateral to the effector. Hence, crossing hands (entering a “foreign” spatial hemifield) yielded an augmented bilateral activation during response monitoring since these two kinds of information were processed in opposing hemispheres. Because the traditional Simon task does not provide the possibility to investigate which aspect of the spatial configuration leads to the observed hemispheric allocation, we introduced a new “double crossed” condition that allows for the dissociation of internal/physiological and external/physical influences on response monitoring processes. Comparing behavioral and neurophysiologic measures of this new condition to those of the traditional Simon task setup, we could demonstrate that the egocentric representation of the physiological effector's spatial location accounts for the observed lateralization of spatial information in action control. The finding that the location of the physical effector had a very small influence on response monitoring measures suggests that this aspect is either less important and/or processed in different brain areas than egocentric physiological information.

## INTRODUCTION

In order to adequately interact with our environment, we constantly monitor our actions so that we can adjust them in case of undesired consequences (Logan, 1985; Stuss and Alexander, 2007; Fukui and Gomi, 2012). Properly doing so is a fairly complex endeavor because for a proper adjustment of the outcome, parameters of movements also need to be integrated in the process of response evaluation.

Given that different features (like speed, spatial position, applied force of the response, etc.) influence our movements, these parameters have to be integrated in the evaluation process (Praamstra et al., 2009; Fukui and Gomi, 2012; Gonzalez and Burke, 2013; Stock et al., 2013). We recently investigated the effects of multimodal sensory integration in response monitoring processes by recording an EEG during a Simon task (see Stock et al., 2013 for details) and demonstrated that both proprioception-based spatial information and efference-copy-based information on the motor execution are integrated in the supplementary motor area (SMA) during response monitoring and evaluation. Among other things, this brain region has been associated with the processing efference copies of motor commands (Neshige et al., 1988; Ikeda et al., 1995; Babiloni et al., 2001; Haggard and Whitford, 2004; Beaul’e, et al., 2012), egocentric proprioceptive information (Tarkka and Hallett, 1991; Hallett, 1994; Loayza et al., 2011), motor control (Angel, 1976; Wolpert and Flanagan, 2001; Allain et al., 2004; Yordanova et al., 2004; Feldman, 2009; Hoffmann and Falkenstein, 2010; Roger et al., 2010), and error monitoring (Peterburs et al., 2011). However, we obtained an unexpected pattern of hemispheric activation by asking the subjects to either cross their hands or keep them parallel while responding: in parallel hands, only the SMA contralateral to the responding hand showed a negative deflection of event-related potentials (ERPs) around the time of the response while the SMA ipsilateral to the responding hand showed a positivation. By contrast, the simple act of crossing one hand one over another reduced most of the differences in hemispheric activation/ERPs as the activity pattern of the hemisphere ipsilateral to the responding hand approximated that of the contralateral hemisphere. This suggests that in case of an unnatural posture (crossed hands) motor efference copies and motor proprioceptive information were allocated to the hemispheres according to different rules: efference-copy-based motor information seemed to be rather immutably locked to the hemisphere in which the motor command was initially processed. In contrast, the hemispheric allocation of proprioception-based spatial information was based on an external representation of space. As a result of these different lateralization mechanisms, crossing hands (manually entering a “foreign” spatial hemifield) most probably resulted in a conflict, yielding an augmented bilateral activation and higher error rates.

Even though these findings seem to answer the question in which hemisphere the monitoring of motor and spatial information is allocated, the paradigm provided no possibility to determine whether the laterlized allocation of spatial information during response monitoring was influenced by internal (proprioceptive) information about the position of the physiologic effector (hand) or by external (egocentric) information about the position of the physical effector (button).

**FIGURE 1:**
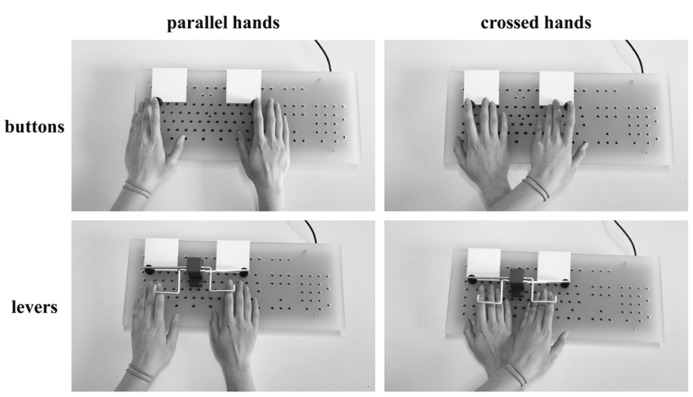
**The four different response conditions resulting from hand position (parallel vs. crossed) and button type (buttons vs. levers).** When crossing hands, the participants were instructed to place the left arm (“marked” with two wristbands in the picture) on top of the right arm. In button responses, the physiological effector (hand) is in the same hemifield as the physical effector (button) so that their relevance for the hemispheric allocation of response monitoring processes cannot be determined. In contrast, the levers provide the necessary dissociation because the physical effector (button) is now located in a different hemifield than the physiological effector (hand). For mechanical reasons, buttons had to be pressed while levers had to be lifted.

In the current study, we aimed at answering this question. For this purpose, we modified the Simon task by introducing a “double crossed” condition. While the regular Simon task only encompasses a parallel-hands and a crossed-hands condition, our new double crossed condition required the subjects to also operate crossed levers in half of the trials. As a consequence, the effect site (button) which was pressed when crossing hands in lever responses was in a different hemifield than the responding hand so that the button was the same as during a regular parallel hands button response (see **Figure 1** for further elucidation). Based on this dissociation of physiological effector (hand) and physical effect site (button), our question could be tackled: in case the spatial allocation of the hand is the relevant factor to the lateralization of response monitoring processes, parallel and crossed hands should yield comparable ERPs, irrespective of whether buttons or levers are used to respond. If however, the external effect site of the button was the critical feature, parallel-hands button responses should yield results similar to those of crossed-hands lever responses.

## MATERIALS AND METHODS

### SAMPLE

Right-handed participants (*N* = 21; 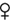 = 11, 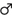 = 10) were included in the study. The mean age was 23.2 years (min 19, max 32, SEM = 0.73) and none of the participants presented with a history of psychiatric or neurological disease. Handedness was confirmed by the Edinburgh Handedness Inventory (Oldfield, 1971), yielding an average score of 0.81 (min 0.25, max 1.0, SEM = 0.05). All subjects gave written informed consent and were treated in accordance with the declaration of Helsinki. Each participant was reimbursed with 15€. The study was approved by the ethics committee of the medical faculty of the University of Bochum.

### SETTINGS AND TASK

Because this study aims to extend previous findings reported by Stock et al. (2013), the settings and task were very similar to that study (see Stock et al., 2013 for details): participants were seated in front of a 17 in CRT computer monitor (at a distance of 57 cm) in a dimly lit and sound-attenuated room. Responses were made with four custom-made buttons mounted on a regular keyboard (see **Figure 1** for illustration).

The Simon task originally references the task used by Wascher et al. (2001). Throughout the whole task, a white fixation cross and two horizontally aligned white frame boxes were continuously displayed in the center of a dark blue screen. The two boxes were at the same vertical level as the fixation cross (1.1° distance between fixation cross and the inner border of the frames). Each trial began with the simultaneous presentation of a target stimulus (a yellow capital letter “A” or “B”) and a noise stimulus (three white horizontal bars). Both target and noise stimuli were approximately 0.5° wide and 0.6° high and presented within the two opposing white boxes. After 200 ms, the stimuli disappeared and the trial was ended by the first (button press) response. If the participants did not respond within the first 500 ms after the onset of the trial, a speed-up sign (containing the German word “Schneller!” which translates to “Faster!”) was presented above the stimuli until the end of the trial. In case no response was given, the trial automatically ended 1700 ms after its onset and was coded as a “miss.” The response–stimulus intervals (RSIs) varied randomly and ranged between 2000 and 2500 ms.

The experiment consisted of eight blocks, each comprising 160 trials. The four stimuli (“A” on the left side/“A” on the right side/“B” on the left side/“B” on the right side) were randomized and occurred equally often, resulting in 40 trials per condition and block. For all blocks, participants were instructed to respond using the left index finger whenever the target stimulus was an “A” and to respond using the right index finger whenever the target stimulus was a “B” (in both cases irrespective of the target’s location on the screen). In blocks 1, 3, 5, and 7 they were asked to respond with parallel hands while they were asked to cross their hands (placing the left arm above the right arm) in blocks 2, 4, 6, and 8. In addition to the setup of our previous study (Stock et al., 2013), participants were requested to respond by pressing the buttons in blocks 1, 2, 5, and 6 while levers had to be used in blocks 3, 4, 7, and 8 (see **Figure 1**). Hence, there were two blocks for each combination of hand position (parallel/crossed) and button type (buttons/levers). Following from this, there were equal numbers of congruent and incongruent trials (classified depending on whether the responding hand was placed in the same hemifield as the target stimulus).

### EEG RECORDING DATA PROCESSING

As for the settings and task, EEG data recording and data processing are very similar to techniques used for our previous publication (see Stock et al., 2013 for details): an EEG was recorded from 65 Ag–AgCl electrodes at standard positions (international 10–20 system) while the participants were performing the task. Electrode impedances were kept below 5 kΩ. During recording, a filter bandwidth of 0–80 Hz was applied and EEG data was recorded with a sampling rate of 1000 samples per second against a reference at electrode FCz. After recording, the data was downsampled to 256 Hz and an IIR filter (notch at 50 Hz; high-pass at 0.5 Hz and low-pass at 18 Hz, using a slope of 48 dB/oct each) was applied. Subsequently, technical artifacts and irregular muscular artifacts (e.g., jaw clenching) were removed during a visual raw data inspection. Uniform artifacts (primarily blinks, eye movements and pulse artifacts) were removed by means of an independent component analysis (ICA) applying the infomax algorithm.

For stimulus-locked event-related lateralizations (ERLs), segments were formed for the different conditions. Epochs started 200 ms before the stimulus presentation (which was set to time point zero) and ended 1200 ms after the response, resulting in a total epoch length of 1400 ms. For the analysis of response-locked event-related potentials (ERPs), segments were formed for the different conditions. Epochs started 1200 ms before the response (which was set to time point zero) and ended 1200 ms after the response, resulting in a total epoch length of 2400 ms.

Independent of the locking point (stimulus or response), only trials that had been correctly answered within the first 1500 ms after the onset of the stimulus presentation were included. Furthermore, an automated artifact rejection removed amplitudes above 100 μV and below -100 μV as well as activity of less than 0.5 μV over a time span of 100 ms or more. Subsequently, a current source density (CSD) transformation was applied to eliminate the reference potential (Perrin et al., 1989; Nunez and Pilgreen, 1991; Nunez et al., 1997).

**FIGURE 2:**
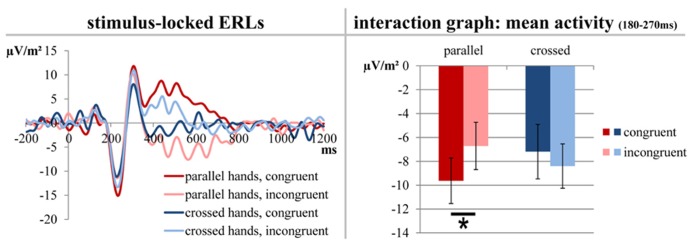
**The stimulus-locked ERLs for electrodes PO7/PO8 obtained by subtracting the ERP curve of the hemisphere ipsilateral to the stimulus presentation site from the ERP curve contralateral to the stimulus presentation site.** Only factors yielding significant results are depicted. The left side of the figure shows the course of the curves; time point zero denotes the onset of stimulus presentation. The right part of the figure elucidates the significant differences found between the mean activity values which average the time span from 180 to 270 ms. The error bars indicate the respective SEMs; significant differences are marked with an asterisk.

For the analysis of stimulus-locked ERLs/N2pc, a baseline correction from -200 to 0 ms was run before the segments of the different conditions were averaged. Based on the topographic distribution of the activity and the literature relevant to this task, ERLs were formed for electrodes PO7 and PO8 (Praamstra and Oostenveld, 2003; Wiegand and Wascher, 2005; Verleger et al., 2012; Cespón, et al., 2013; Stock et al., 2013). For this purpose, the values of the hemisphere ipsilateral to the target stimulus site were subtracted from the values of the hemisphere contralateral to the target stimulus site (PO7–PO8 for stimuli presented on the right side and PO8–PO7 for stimuli presented on the left side) and averaged for both hands. For statistical analyses, we extracted the mean electrode activity between 180 and 270 ms (the time frame was based on the negative peak and differences between the conditions; see **Figure 2**).

**FIGURE 3:**
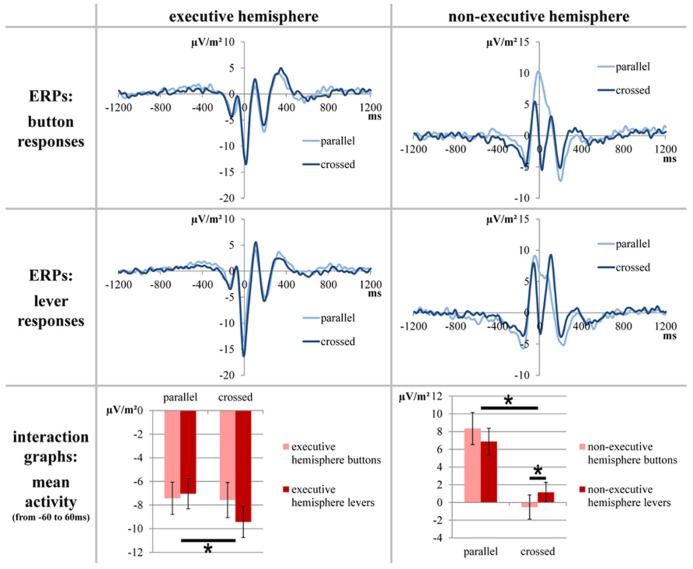
**The stimulus-locked ERPs for electrodes FC1/FC2.** Only factors yielding significant results are depicted. The upper parts of the figure show the course of the curves; time point zero denotes the response. The lower part of the figure elucidates the significant differences found between the mean activity values which average the time span from -60 to 60 ms. The error bars indicate the respective SEMs; significant differences are marked with an asterisk.

For the analysis of response-locked ERPs, a baseline correction from -1200 to -800 ms was run before the segments of the different conditions were averaged. Based on our previous study, we decided to quantify the response-locked ERPs at electrodes FC1 and FC2 because these electrodes have been shown to optimally depict response evaluation differences/changes in SMA activity between the different conditions of this task (see Coles, 1989; Leuthold, 2011; Stock et al., 2013 for details). For statistical analyses, we extracted the mean electrode activity between -60 and 60 ms (the time frame was based on the differences between the conditions; see **Figure 3**).

### STATISTICAL ANALYSIS

Behavioral data (RTs and the number of hits/correct responses) were analyzed using repeated-measures analyses of variance (ANOVA). “Button type” (button responses vs. lever responses), “hand position” (parallel hands vs. crossed hands), and “congruency” (congruent vs. incongruent; codes whether the target stimulus was presented on the side where the responding hand was placed) were used as within-subjects factors. The electrophysiological stimulus-locked data was analyzed using repeated-measures ANOVA with the within-subjects factors “button type,” “hand position,” and “congruency.” Because ERLs are based on the difference between the hemisphere contralateral and ipsilateral to the stimulus presentation site, there was no factor for side/hemisphere. The electrophysiological response-locked data was analyzed in similar fashion using a repeated-measures ANOVA with the within-subjects factors “button type,” “hand position,” “congruency,” and “executive hemisphere” (electrode above the hemisphere responsible for the motor execution of the response vs. electrode above the hemisphere irresponsible for the motor execution of the response). Greenhouse–Geisser-correction was used whenever necessary. All *p*-levels for *post hoc* *t*-tests were adjusted using Bonferroni correction. Effect sizes were given as the proportion of variance accounted for (η ^2^ ). As a measure of variability, the standard error of the mean (SEM) together with the mean values was given.

## RESULTS

### BEHAVIORAL DATA

#### Accuracy

A repeated-measures ANOVA of the percentage of hits (within-subjects factors “button type,” “hand position,” and “congruency”) revealed a significant main effect for “hand position” [*F*(1,20) = 4.571; *p* = 0.045; η ^2^ = 0.186] with more correct answers in parallel-hands trials (89.0% ± 1.653) than in crossed-hands trials (86.6% ± 1.506). There was also a significant main effect for “congruency” [*F*(1,20) = 1.197; *p* < 0.001; η ^2^ = 0.792] with more correct answers in congruent trials (91.8% ± 1.336) than in incongruent trials (83.7% ± 1.735). There was also a significant interaction of “button type” × “congruency” [*F*(1,20) = 19.845; *p* < 0.001; η ^2^ = 0.498]. *Post hoc* *t*-tests revealed that buttons yielded more correct responses than levers in congruent trials [*t*(20) = 2.255; *p* = 0.036; buttons: 94.5% ± 0.695 and levers: 89.2% ± 2.434] but not in incongruent trials [*t*(20) = -0.217; *p* = 0.831]. Furthermore, there was a significant interaction of “hand position” × “congruency” [*F*(1,20) = 9.691; *p* = 0.005; η ^2^ = 0.326]. *Post hoc* *t*-tests revealed that there were more correct answers in parallel-hands trials than in crossed-hands trials only in incongruent trials [*t*(20) = 3.163; *p* = 0.005; parallel: 86.0% ± 1.904 and crossed: 81.5% ± 1.848] but not in congruent trials [*t*(20) = 0.262; *p* = 0.796].

#### Response times

A repeated-measures ANOVA of the RTs of correct responses (within-subjects factors “button type,” “hand position,” and “congruency”) revealed significant main effects for all three factors: “hand position” significantly differed [*F*(1,20) = 7.365; *p* = 0.013; η ^2^ = 0.269] with correct parallel-hands response being faster (442.4 ms ± 9.579) than correct crossed-hand responses (452.1 ms ± 10.247). There was also a significant main effect for “button type” [*F*(1,20) = 27.783; *p* < 0.001; η ^2^ = 0.581] with correct button responses being faster (436.1 ms ± 8.958) than correct lever responses (458.4 ms ± 10.914). The significant main effect for “congruency” [*F*(1,20) = 73.787; *p* < 0.001; η ^2^ = 0.787] was based on faster responses in congruent trials (435.8 ms ± 10.048) than in incongruent trials (458.7 ms ± 9.643). There were also a significant interaction of “button type” × “congruency” [*F*(1,20) = 29.994; *p* < 0.001; η ^2^ = 0.600] and a significant threefold interaction of “hand position” × “button type” × “congruency” [*F*(1,20) = 7.547; *p* = 0.012; η ^2^ = 0.274]. A *post hoc* repeated-measures ANOVA confined to lever responses only showed a significant main effect for “congruency” [*F*(1,20) = 21.492; *p* < 0.000; η ^2^ = 0.518] with RTs in congruent trials being faster (450.9 ms ± 11.724) than RTs in incongruent trials (465.8 ms ± 10.294). In contrast, the *post hoc* repeated measures ANOVA confined to the button responses found a significant main effect for “congruency” [*F*(1,20) = 117.445; *p* < 0.001; η ^2^ = 0.854; congruent: 420.6 ms ± 8.632 and incongruent: 451.5 ± 9.490] as well as for “hand position” [*F*(1,20) = 9.285 *p* = 0.006; η ^2^ = 0.316; parallel: 428.7 ms ± 8.614 and crossed: 443.4 ± 9.902]. However, none of the ANOVAs showed a significant interaction (*p* ≥ 0.129).

#### Summary of behavioral results

Briefly summing up the behavioral results, significant interactions show that the subjects hit rate was differently modulated across congruency: in congruent trials only, button responses had higher hit rates than lever responses while in incongruent trials only, parallel-hand responses had higher hit rates than crossed-hand responses.

Furthermore, a threefold interaction indicated that hit RTs were modulated by button type, congruency, and hand position: while congruency modulated the RT in both button and lever responses (congruent faster than incongruent), only button response RTs were additionally modulated by hand position (parallel faster than crossed).

### NEUROPHYSIOLOGICAL DATA

#### Stimulus-locked data

Stimulus-locked data at electrodes PO7 and PO8 are depicted in **Figure 2**.

A repeated measures ANOVA (within-subjects factors “button type,” “hand position,” and “congruency”) of the mean ERL activity at electrodes PO7 and PO8 (stimulus-locked data; averaged from 180 to 270 ms) was run. It yielded a significant interaction of “hand position” × “congruency” [*F*(1,20) = 7.968, *p* = 0.011, η ^2^ = 0.285]. *Post hoc* *t*-tests showed that congruent trials produced a bigger/more negative ERL (-9.629 μV/m^2^ ± 1.913) than incongruent trials (-6.712 μV/m^2^ ± 1.980) in parallel-hand trials [*t*(20) = -3.669, *p* = 0.002] but not in crossed-hand trials [*t*(20) = 1.301, *p* = 0.208; see **Figure 2** for visualization].

#### Response-locked data

Response-locked ERPs at electrodes FC1 and FC2 are depicted in **Figure 3**.

A repeated measures ANOVA (within-subjects factors “button type,” “hand position” “executive hemisphere,” and “congruency”) of the mean activity at electrodes FC1 and FC2 (response-locked data; averaged from -60 to 60 ms) was run. It yielded a significant main effect for “hand position” [*F*(1,20) = 43.474; *p* < 0.001; η ^2^ = 0.685] with a positive mean activity for correct parallel-hands responses (0.189 μV/m^2^ ± 1.296) and a negative mean activity for correct crossed-hands responses (-4.094 μV/m^2^ ± 1.197). The main effect for “executive hemisphere” was also significant [*F*(1,20) = 189.227; *p* < 0.001; η ^2^ = 0.904] with a negative mean activity over the executive hemisphere (-7.867 μV/m^2^ ± 1.236) and a positive mean activity over the non-executive hemisphere (3.962 μV/m^2^ ± 1.321) during correct responses. There was a significant interaction for “hand position” × “congruency” [*F*(1,20) = 5.220; *p* = 0.033; η ^2^ = 0.207]. However, this interaction did not survive *post hoc* testing. *Post hoc* *t*-tests revealed that congruent and incongruent trials neither differed in the parallel-hands condition [*t*(20) = -1.869; *p* = .076] nor in the crossed-hands condition [*t*(20) = 1.523; *p* = 0.143]. Likewise, there were significant differences between hand positions in both congruent [*t*(20) = 4.775; *p* < 0.001] and incongruent trials [*t*(20) = 5.957; *p* < 0.001]. Furthermore, there was a significant interaction for “hand position”×“executive hemisphere” [*F*(1,20) = 61.960; *p* < 0.001; η ^2^ = 0.756]. Finally, there was a significant threefold interaction for “hand position” × “executive hemisphere” × “button type” [*F*(1,20) = 35.912; *p* < 0.001; η ^2^ = 0.642]. A *post hoc* repeated-measures ANOVA confined to the executive hemisphere only showed significant main effect for hand position [*F*(1,20) = 5.760; *p* = 0.026; η ^2^ = 0.224] with parallel hands evoking a smaller mean amplitude (-7.233 μV/m^2^ ± 1.217) than crossed hands (-8.500 μV/m^2^ ± 1.308) in correct responses. The *post hoc* repeated measures ANOVA confined to the non-executive hemisphere found a significant main effect for “button type” [*F*(1,20) = 62.058; *p* < 0.001; η ^2^ = 0.756; buttons: 3.912 μV/m^2^ ± 1.504 and levers 4.012 μV/m^2^ ± 1.232] and significant interaction of “button type” × “hand position” [*F*(1,20) = 10.191 *p* = 0.005; η ^2^ = 0.338]. *t*-Tests revealed that in the non-executive hemisphere, there was a difference between button types for correct crossed-hand responses [*t*(20) = -2.331; *p* = 0.030 with buttons -0.522 μV/m^2^ ± 1.368 and levers 1.149 μV/m^2^ ± 1.119] but not for parallel-hand responses [*t*(20) = 1.384; *p* = 0.182; see **Figure 3** for visualization].

#### Summary of neurophysiological results

Briefly summing up the electrophysiological results, the stimulus-locked ERLs of correct responses were modulated by an interaction of congruency and hand position: only in parallel-hand responses, congruent trials evoked significantly more negative ERLs than incongruent trials. Furthermore, the response-locked ERPs of correct responses were modulated by an interaction of button type, hand position, and hemisphere (but not by congruency): in the non-executive hemisphere, button and lever responses differed from each other when hands were crossed (but not when they were parallel). By comparison, the mean amplitudes of the executive hemisphere only differed between parallel and crossed-hand responses.

## DISCUSSION

The current study aimed at determining whether the location of an internal/physiologic effector (hand) or the location of an external, physical effector (response button) accounts for the previously observed asymmetric lateralization of spatial aspects of response monitoring processes (Stock et al., 2013).

The results (especially the interaction pattern observed in the response-locked ERP data) suggest that the spatial location of the physiologic effectors accounts for the largest part of the observed changes in the hemispheric allocation of spatial information during response monitoring. In order to elucidate the rationale behind this interpretation, we would like to explain the theoretical background of our experimental manipulation: the basic assumption behind the additional factor “button type” is that “each hemisphere preferentially processes and integrates the contralateral egocentric and allocentric spatial information” (Zhou et al., 2012). Following from this, trials with button responses provide a “baseline” measurement because the hand and button involved in a response are always located in the same spatial hemifield. Differences between the two hand positions (parallel vs. crossed) can be attributed to spatial properties of the effectors, but the effectors (hand vs. button) cannot be told apart. In contrast to this, trials with lever responses provide the measurement of our “experimental manipulation” because in this condition, the responding hand and the button pressed are always located in opposing spatial hemifields. Hence, the influence of the different effectors can be distinguished by comparing baseline and experimental manipulation/button and lever trials: influences exerted by the physiologic effector/the location of the hand should yield identical or at least similar result for both button types (i.e., parallel-hand button responses ≈ parallel-hand lever responses and crossed-hand button responses ≈ crossed-hand lever responses). In contrast to this, influences exerted by the physical effector/the location of the button should yield opposing or at least different results for the two button types (i.e., parallel-hand button responses ≈ crossed-hand lever responses and crossed-hand button responses ≈ parallel-hand lever responses).

The first option is basically what was observed in the response-locked ERPs. Such fronto-central ERPs are known to reflect response monitoring and evaluation processes and are most likely generated within the SMA, anterior cingulate cortex, and adjacent areas (Macar et al., 1999; Luu and Tucker, 2001; Beste et al., 2010a, b, 2012; Roger et al., 2010; Wascher and Beste, 2010). In our previous study, we could demonstrate the response-locked ERPs quantified in this study originate within the SMA and are sensitive to the spatial allocation of the effector (Stock et al., 2013). As described above, we aimed at identifying the effector (physical or physiological) by comparing button and lever response conditions. As can be seen in the top row (“button responses”) of **Figure 3**, placing the effectors in their usual hemifield yields a positivation of the response-locked ERP over the non-executive hemisphere. By contrast, placing the effectors in the “foreign” hemifield yields a negativation of the response-locked ERP over the non-executive hemisphere so that it resembles the course of the ERP curve over the executive hemisphere. Furthermore, it can be noted that the ERP over the non-executive hemisphere is more negative when the effectors are placed in the contralateral hemifield. A repeated-measures ANOVA was run to compare lever responses to button responses. Due to the interactions of factors, the main effects of hand position and hemisphere cannot be subject to interpretation. We would however like to point out that there was no main effect of button type. Hence, there was no basic fundamental difference between buttons and levers which is in favor of assuming the hands to be the relevant effectors. Two interactions are important: first, there was an interaction of hand position and congruency. Because both *post hoc* tests yielded significant differences between the hand positions (each parallel > crossed), the finding only differed quantitatively between congruent and incongruent trials. Second, there was a threefold interaction of button type, hand position, and hemisphere. This interaction is crucial when trying to answer the question of which effector (hand or button) accounts for lateralization of spatial aspects of response monitoring processes. The button type had no effect on the executive hemisphere that always processes efference-copy-based information of the motor aspects of the response and information on spatial properties of the response in half of the trials. In the non-executive hemisphere, the button type only had an effect in crossed hands (lever responses yielding more positive ERPs than button responses), but not in parallel hands.

Our interpretation is as follows: the fact that the activation of the non-executive hemisphere does not differ in parallel-hand responses suggests that this hemisphere does not contribute to response monitoring/process spatial information in neither button nor lever response trials. This suggests that the location of physiological effectors (the hands which stayed within their “natural” hemifield) accounts for the lateralization of response monitoring processes and that the physical effector (the location of the button) does not. The non-executive hemisphere difference between buttons and levers in crossed hands is not strictly in line with the assumption that only the hands are responsible for the hemispheric allocation of spatial information during response monitoring. Yet, it is unlikely that the physical effector (button) plays a major role in the allocation of response monitoring processes. The reason for this is that based on the explanations above, one would expect a “reversal” of parallel and crossed non-executive hemisphere ERPs across the button types. In case of an allocation based on the location of the physical effector, lever responses should produce a positive peak in crossed hands and a negative peak in parallel hands (crossed > parallel) over the non-executive hemisphere. This criterion is not fulfilled since both in button and in lever responses; parallel hands yield a more positive ERP than crossed hands (see **Figure 1**). Because of the different polarity of ERP peaks around the time of the response, we based the statistical analysis on mean activity measures. While these measures can depict differences between the epochs over which the ERP data was averaged, they unfortunately cannot account for the course of the curves within these epochs. Yet, we obtained no convincing statistical results in favor of a physical effector approach and the grand averages (**Figure 3**) further support the assumption that the physiologic effector (hand) determines the allocation of spatial response monitoring processes: despite the detected differences, the course of the ERP curves of crossed-hand lever responses is very similar to that of crossed-hand button responses while both crossed-hand conditions markedly differ from the course of parallel-hand responses.

Furthermore, the behavioral results of this study are line with previous findings on this paradigm (e.g., Wiegand and Wascher, 2005; Leuthold, 2011) suggesting that the task was correctly implemented/worked as intended. Both hit rates and RTs were modulated by the hand position as well as the spatial congruency of the stimulus presentation site and the location of the responding hand. In all significant main effects and interactions, parallel-hand responses yielded a better (more accurate/faster) performance than crossed-hand responses and congruent trials yielded better results than incongruent trials. Matching results were obtained for the stimulus-locked ERLs/N2pc. As expected, the ERLs showed an interaction of hand position and congruency (see Praamstra and Oostenveld, 2003; Wiegand and Wascher, 2005; Böckler et al.,2011; Leuthold, 2011; Verleger et al., 2012). For the ERLs, there was no effect of button type whatsoever. Since stimulus–response congruency had been defined with respect to the location of the hand (not the button), this finding clearly indicates that external/physical effectors do not seem to have an influence on congruency and on early attentional processing and/or filtering (Luck and Hillyard, 1994; Böckler et al., 2011; Leuthold, 2011; Verleger et al., 2012).

From this study, it can be concluded that the spatial location of physiologic effectors (in our case, this would be the hands) plays a major role in the asymmetrical allocation of response monitoring processes: whenever the physiologic effectors enter a “foreign” hemifield, the hemisphere contralateral to this hemifield seems to handle information on spatial aspects of the response. By comparison, the location of the physical effector (in our case, this would be the buttons) plays a minor role. Yet, the possibility that it still contributes to response monitoring processes cannot be ruled out completely. Furthermore, these findings allow for the conclusion that potentially different action goals of button and lever responses do not substantially influence the lateralized allocation of response monitoring processes (compare to Buhlmann et al., 2007). Our study extends the established fact that each hand operates “in its own egocentric space” (Haggard et al., 2000) by demonstrating that egocentric space continues to play a role in the subsequent processes of response monitoring and evaluation. Also, our results are in line with the findings that proprioceptive (Allain et al., 2004) and internal sensorimotor information is used for response evaluation (Fukui and Gomi, 2012) and that each hemisphere preferentially processes information from the contralateral hemifield (Zhou et al., 2012).

## AUTHOR CONTRIBUTIONS

Both authors contributed to the design of the experiment, data collection, the interpretation of results, and to the written manuscript.

## Conflict of Interest Statement

The authors declare that the research was conducted in the absence of any commercial or financial relationships that could be construed as a potential conflict of interest.
